# Modifiable factors for migraine prophylaxis: A mendelian randomization analysis

**DOI:** 10.3389/fphar.2023.1010996

**Published:** 2023-01-12

**Authors:** Hui Zheng, Yun-Zhou Shi, Jing-Tao Liang, Liang-Liang Lu, Min Chen

**Affiliations:** ^1^ The Third Hospital/Acupuncture and Tuina School, Chengdu University of Traditional Chinese Medicine, Chengdu, China; ^2^ Department of Neurology, Hospital of Chengdu University of Traditional Chinese Medicine, Chengdu, China; ^3^ Department of Neurology and Rehabilitation, Deyang Jingyang Hospital of Traditional Chinese Medicine, Deyang, China; ^4^ Department of colorectal diseases, Hospital of Chengdu University of Traditional Chinese Medicine, Chengdu, China

**Keywords:** modifiable risk factors, migraine prophylaxis, mendelian randomization, public health, schooling

## Abstract

**Objective:** To examine the causal effect of potentially modifiable risk factors contributing to migraine pathogenesis.

**Methods:** We performed Mendelian randomization analyses and acquired data from United Kingdom Biobank, FinnGen Biobank, and the MRC IEU OpenGWAS data infrastructure. An inverse-variance weighted (IVW) model was used to examine the relationship between 51 potentially modifiable risk factors and migraine in 3215 participants with migraine without aura (MwoA), 3541 participants with migraine with aura (MwA), and 176,107 controls. We adopted a Bonferroni-corrected threshold of *p* = 9.8 × 10–4 (.05 divided by 51 exposures) as a sign of significant effect, and a *p* < .05 was considered as the sign of a suggestive association.

**Results:** More years of schooling significantly correlated with lower odds of MwoA pathogenesis (OR .57 [95%CI .44 to .75], *p* < .0001). More vitamin B12 intake (OR .49 [95%CI .24 to .99], *p* = .046) and lower level of stress [OR 8.17 (95%CI 1.5 to 44.36), *p* = .015] or anxiety disorder (OR 1.92 × 109 [95%CI 8.76 to 4.23*1017], *p* = .029) were suggestive to be correlated lower odds of MwoA pathogenesis. More coffee intake (OR .39 [95%CI .22 to .7], *p* = .001), lower level of eicosapentaenoic acid status (OR 2.54 [95%CI 1.03 to 6.26], *p* = .043), and more light physical activity (OR .09 [95%CI .01 to .94], *p* = .046) were suggestive to be associated with lower odds of MwA.

**Conclusion:** The years of schooling, light physical activity, vitamin B12 intake, and coffee intake were the protective factors for migraine; stress, anxiety, and eicosapentaenoic acid status were harmful factors. Interventions could be developed based on modifying these factors for migraine prophylaxis.

## Highlights

• Potentially modifiable risk factors for migraine pathogenesis are unclear. The study addressed which are the modifiable risk factors contributing to migraine attacks.
• Based on Mendelian randomization analyses, we found that more years of schooling, vitamin B12 intake, and light physical activity were correlated with lower odds of migraine, while lower levels of stress, anxiety disorder, and eicosapentaenoic acid status were associated with lower odds of migraine.
• Comprehensive interventions could be developed based on modifying the years of schooling, vitamin B12 intake, light physical activity, levels of stress, anxiety disorder, and eicosapentaenoic acid status for migraine prevention.

## Introduction

Migraine is a highly prevalent neurological disorder, characterized by frequent headache attacks accompanying photophobia, phonophobia, nausea, or vomiting. It is reported that migraine has a global prevalence of 10%, which brings a heavy burden on healthcare expenditure and becomes a major public health problem.

The management of migraine includes lifestyle modifications, pharmacological treatments, non-pharmacological treatments, and complementary and alternative therapies. Pharmacological treatments are recommended by guidelines and commonly practiced in clinical settings. However, the effectiveness of the pharmacological treatments varies in differential drug classes, and many of the treatments have low response rates for migraine prophylaxis (Shamliyan et al., 2013). The reason for the variation in the treatment effects is that most of the pharmacological treatments are not specifically developed for the preventive treatment of migraine. For example, calcium channel blockers and βblockers are recommended as first-line treatments for migraine prophylaxis, and they are developed for hypertension. High-quality evidence is still lacking for non-pharmacological treatments and complementary and alternative therapies, hampering their generation in practice.

Regarding the unmet treatment needs for migraine management, it is proposed that clinicians should use evidence-based multidisciplinary approaches to optimize clinical practices (Ashina et al., 2021). Clarifying the modifiable risk factors that are correlated with the pathogenesis of migraine is essential for developing multidisciplinary strategies for migraine prophylaxis and individualized approaches for patients with migraine. Many risk factors have been reported in recent 20 years, and most of them were revealed through observational studies. Owing to the disadvantages of the observational design, these findings are susceptible to confounding bias and reverse causation (Yarmolinsky et al., 2018; Hammerton and Munafò, 2021). Mendelian randomization, using genetic instruments as probes to detect causal relationships between exposures and disease outcomes, is perceived as a new study design that can minimize the risk of confounding bias and reverse causation. The nature of the randomized assignment of genetic effect alleles makes a Mendelian randomization study analog to a randomized trial (Davies et al., 2018). No study has been conducted to systematically search for potential risk factors that have causal effects on the pathogenesis of migraine, although several MR studies have fragmentarily explored a few of them.

We performed a comprehensive search for potentially modifiable risk factors that are reported in previous reviews or studies and implemented MR analyses to clarify whether the factors have causal effects on migraine pathogenesis.

## Materials and methods

We acquired deidentified summary-level data from large-scale genome-wide association studies (GWASs). The data were openly available, and the GWAS studies had ethical approval from the participating centers. The design and conduct of this Mendelian randomization study conformed to the STROBE-MR (Strengthening the Reporting of Observational Studies in Epidemiology Using Mendelian Randomization) statement (Skrivankova et al., 2021).

### Exposure data

Before the conduct of our MR study, we performed a literature search in Medline, looking for systematic reviews, meta-analyses, reviews with evidence summary, or studies in recent 3 years of modifiable factors for the prevention of migraine, using the keyword: migraine, risk factors, association factors, lifestyle, diet, physical activity, sleep, supplements, vitamin, and metabolism. We read the retrieved articles and screened for potentially modifiable risk factors that were associated with migraine. The reference articles were provided in Supplementary eTable S1.

Fifty-one risk factors were finally identified after the literature review. Nine factors were diet-related; eighteen were dietary supplements or serum indexes-related factors; thirteen were lifestyle-related; eleven were cardio-metabolic factors. The factors were listed in Table 1. Correlated SNPs of each factor were identified from the MRC IEU OpenGWAS data infrastructure (Hemani et al., 2018; Elsworth et al., 2020), which included a database of 214,846,534,918 genetic associations from 39,994 GWAS summary datasets. Each exposure factor and its related SNPs were further confirmed by reading the corresponding article IDs provided in the database.

**TABLE 1:** Summary of modifiable risk factors for migraine in the Mendelian randomization analysis.

Modifiable risk factors	Number of SNPs used in the analysis	The proportion of variance explained by SNPs (%)	The effect size of the SNPs on the risk factors (beta, se)		F Statistics	I2 GX (%)	Data source
			Migraine without aura	Migraine with aura			
Diet							
Alcohol intake frequency	42	8.16	19 (.19)	−041 (.158)	110.7	98.3	United Kingdom biobank
Alcoholic drinks per week	34	4.82	−25 (.32)	−06 (.328)	165.9	98.7	PMID30643251
Average weekly red wine intake	18	5.39	−62 (.52)	−684 (.533)	52.1	97.7	United Kingdom biobank
Coffee intake	40	4.78	−458 (.312)	−933 (.292)	54.5	98.5	United Kingdom biobank
Decaffeinated coffee intake	14	1.22	−472 (.523)	−078 (.402)	24.3	90.0	United Kingdom biobank
Carbohydrate	19	4.11	−17 (.23)	−008 (.271)	22.9	95.7	United Kingdom biobank
Process meat	23	4.35	−16 (.5)	−238 (.436)	36.4	97.4	United Kingdom biobank
Cheese intake	61	6.67	11 (.26)	−078 (.281)	37.5	97.3	United Kingdom biobank
Saturated fat intake	11	3.2	−23 (.31)	007 (.307)	23.4	95.5	United Kingdom biobank
Polyunsaturated fat intake	15	1.85	−16 (.25)	−362 (.215)	22.4	95.5	United Kingdom biobank
Total sugar intake	18	4.96	−25 (.21)	−01 (.212)	22.8	95.5	United Kingdom biobank
Dietary supplements or metabolites							
Blood methionine	13	NA	03 (1.16)	−306 (1.106)	22.4	95.5	PMID24816252
Blood selenium	12	NA	1.78 (2.52)	−2.11 (2.828)	18	93.2	United Kingdom biobank
Blood zinc	7	NA	06 (.07)	−024 (.076)	29.1	94.8	PMID23720494
Vitamin D intake	14	.68	6.051 (4.072)	4.179 (3.851)	21.9	95.8	United Kingdom biobank
Carotene intake	16	2.97	−038 (.246)	−104 (.24)	22.7	95.2	United Kingdom biobank
Iron intake	13	4.46	−026 (.292)	−334 (.303)	23.2	95.9	United Kingdom biobank
Calcium intake	19	4.32	−03 (.233)	047 (.245)	22.9	95.6	United Kingdom biobank
Folate intake	14	4.15	028 (.233)	203 (.221)	22.3	95.9	United Kingdom biobank
Vitamin B6 intake	16	3.6	−08 (.347)	−177 (.25)	22.1	95.9	United Kingdom biobank
Vitamin B12 intake	9	1.87	−721 (.362)	.320 (.292)	22	94.6	United Kingdom biobank
Vitamin E intake	11	3.45	−21 (.245)	−258 (.352)	23.5	95.5	United Kingdom biobank
Omega-3 status	49	2.87	018 (.061)	022 (.062)	122.5	99.6	PMID27005778
Omega-6 status	53	5.04	−054 (.083)	−077 (.088)	106.4	99.1	PMID27005778
Omega-7, omega-9 and saturated fatty acids	7	2.5	07 (.104)	054 (.099)	52.7	97.7	PMID27005778
Eicosapentaenoic acid status	7	1.2	774 (.484)	933 (.46)	24.4	97.3	PMID24816252
Docosahexaenoic acid status	35	1.68	−418 (.268)	074 (.286)	18.8	94.7	PMID24816252
Lifestyle factors							
Years of schooling	305	10.65	−554 (.136)	−149 (.12)	46.4	98.0	PMID30038396
Stress owing to financial difficulties	86	3.38	2.1 (.863)	1.227 (.868)	23.6	95.8	United Kingdom biobank
Anxiety disorder	17	.11	21.378 (9.8)	−6.271 (9.669)	22.6	93.4	United Kingdom biobank
Depression	33	1.59	1.433 (2.256)	2.671 (1.878)	22.6	95.8	United Kingdom biobank
Sleep duration	69	7.02	−333 (.334)	−088 (.292)	42.3	97.7	United Kingdom biobank
Night shift work	7	1.14	297 (.291)	221 (.352)	22.2	96.2	United Kingdom biobank
Snoring	41	5.94	−486 (.643)	926 (.69)	39.7	97.5	United Kingdom biobank
Insomnia	39	6.14	143 (.421)	2 (.419)	52.4	97.8	United Kingdom biobank
Years of smoking	10	9.55	238 (.273)	4 (.298)	68.4	98.6	United Kingdom biobank
Heavy physical activity	17	3.46	−799 (1.34)	−614 (1.261)	34.6	97.1	United Kingdom biobank
Light physical activity	12	3.77	−2.182 (1.225)	−2.43 (1.211)	37.6	97.5	United Kingdom biobank
Number of days/weeks of vigorous physical activity	11	3.58	−.386 (.305)	.078 (.32)	39.5	97.5	United Kingdom biobank
Number of days/weeks of moderate physical activity	18	4.01	−186 (.215)	−319 (.215)	35.4	97.3	United Kingdom biobank
Cardio-metabolic parameters							
Body mass index	432	23.32	−16 (.082)	−1 (.081)	50.2	98.4	United Kingdom biobank
Waist-to-hip ratio	30	9.83	−123 (.212)	−214 (.216)	46.3	97.9	PMID25673412
Fasting glucose	30	37	−128 (.224)	101 (.227)	102.9	99.3	PMID22885924
Fasting proinsulin	8	NA	−02 (.137)	−035 (.13)	71.3	98.9	PMID21873549
Hemoglobin A1c	11	6.12	−465 (.41)	319 (.367)	71.7	98.7	PMID20858683
Systolic blood pressure	437	12.84	−004 (.005)	008 (.005)	71	98.7	PMID30224653
Diastolic blood pressure	437	12.84	−016 (.009)	012 (.008)	74.7	98.7	PMID30224653
High-density lipoprotein	326	10.32	−092 (.063)	−103 (.059)	174	99.4	PMID32203549/United Kingdom Biobank
Low-density lipoprotein	158	3.12	−08 (.074)	−096 (.077)	228.5	99.4	PMID32203549/United Kingdom Biobank
Total cholesterol	84	10.89	−095 (.067)	−012 (.067)	178.2	99.3	PMID24097068
Total triglycerides	12	9.94	−059 (.093)	05 (.076)	69	98.3	PMID27005778

Beta, The effect size of the exposure on MDD. IVW, inverse variance weighted. MR, Mendelian randomization. Se, standard error. SNP, single nucleotide polymorphisms.

Most of the exposure factors were observed in the United Kingdom Biobank, which is a prospective cohort study with deep genetic, physical, and health data collected on ∼500,000 individuals across the United Kingdom from 2006 to 2010 (Bycroft et al., 2018). The other exposures were extracted from 12 GWAS studies, of which the PMIDs were listed in Table 1. We described the number of SNPs for each exposure trait and the pooled effect size of the SNPs on the trait.

### Selection of instrumental variables

The instrumental variables (IVs), normally refer as SNPs in the Mendelian randomization studies, were selected according to predefined criteria. First, we selected SNPs with a correlation *p*-value <5×10^8^ in the reported GWAS studies. In the traits that had no SNP surviving the *p*-value limit, we relaxed the *p*-value to <1×10^6^ according to previous MR studies. Second, we clumped the data with an *r* ^2^ threshold of .001 and a distance window of 10,000 kB for minimizing bias from linkage disequilibrium (LD). Third, we harmonized the exposure data and the outcome data by removing SNPs for incompatible alleles or SNPs for being palindromic with intermediate allele frequencies, and we finally obtained a dataset containing data for both exposure traits and migraine outcomes which was used for Mendelian analysis.

### Migraine outcomes

The outcome data, the summary-level genomic data of migraine, was acquired from the FinnGen study, which was launched in Finland in 2017. The FinnGen study, also known as Finn biobank, provides genome information and digital healthcare data of 506,800 participants (https://www.finngen.fi/en). We included 3215 participants with migraine without aura (MwoA), 3541 participants with migraine with aura (MwA), and 176,107 participants as controls. The participants with migraine were diagnosed with the International Classification of Diseases criteria (eighth version, code 346; ninth version, code 346; tenth version, code G43). These participants were of European ancestry, and both males and females were included. The data were released in the FinnGen biobank analysis round 5, and we imported the data from the MRC IEU OpenGWAS data infrastructure.

### Statistical analysis

Using a two-sample MR analysis, we estimated the causal effects of each exposure (risk factor) on migraine, utilizing the inverse variance weighted (IVW) method. We extracted the SNP-exposure association (X) and SNP-migraine association estimates from the respective GWAS study, calculated the ratio of Y *versus* X for each SNP, and combined the ratios of all SNPs extracted for a specific exposure using the IVW method. For exposures reported with continuous data (eg, alcoholic intake frequency, years of schooling, and BMI), the combined estimates were explained as the log-odds of developing migraine with per genetically predicted standard deviation (SD) difference in the exposure. For exposures reported with binary data (eg, snoring, insomnia, and depression), the combined estimates were explained as the odds ratios (ORs) per genetically predicted unit difference in log-odds of having the relevant exposures. We drew scatter plots to show the direction of the causal effect and forest plots to show the effect size and whether the effect was statistically significant. To adjust for multiple comparison bias, we adopted a Bonferroni-corrected threshold of *p* = 9.8 × 10^−4^ (.05 divided by 51 exposures) as a sign of significant effect, while a *p* < .05 was considered as the sign of a suggestive association.

We performed heterogeneity tests using Cochran’s Q-statistic to downweigh or exclude SNPs with heterogeneous causal estimates. The Q-statistic is based on the first-order weights of the IVW model which assumes that there is no measurement error (NOME) in the genetic associations with the risk factor (Rees et al., 2019). We then performed a leave-one-out analysis to detect whether the causal effect of exposure was led by one dominant SNP.

We performed several sensitivity analyses to minimize confounding issues. First, we performed MR-Egger analysis, a meta-regression of the estimates of SNP-outcome association on the estimates of SNP-exposure association, to examine whether there was directional pleiotropy. We would assume no directional pleiotropy in the MR analysis when the intercept of the MR-Egger regression approached zero and the results were consistent between the IVW and the MR-Egger analysis. Second, we implemented the weighted median analysis, which had greater robustness than the IVW and the MR-Egger methods in handling individual genetic variants with strongly outlying causal estimates (Bowden and Holmes, 2019). Third, we carried out IVW radial analysis, an analysis that adopts a range of weighting specifications and determines outliers concerning their contribution to global heterogeneity. In the IVW radial model, a significance threshold of .05 and a tolerance threshold of .0001 were selected. Fourth, we implemented the Mendelian Randomization Pleiotropy RESidual Sum and Outlier (MR-PRESSO) test, which evaluates overall horizontal pleiotropy by comparing the observed distance of all the SNPs to the regression line (residual sum of squares) with the expected distance under the null hypothesis of no horizontal pleiotropy (Verbanck et al., 2018). We provided the results of the MR-PRESSO global test, and outlier-corrected estimates would be provided when there were indications of horizontal pleiotropy.

To assess weak instrument bias, we estimated the F-statistics for each exposure by using the method developed for two-sample MR (13). Larger F-statistic values indicate a stronger instrument variable, and we used a threshold of 10 to determine whether there was weak instrument bias (Burgess and Thompson, 2011). It is reported that F-statistic is not sufficient for evaluating weak instrument bias in the MR-Egger regression analysis, so we also reported I2 GX for each exposure. A high value of I2 GX, larger than 90%, indicates that weak instrument bias is unlikely to affect the MR-Egger analysis (Bowden et al., 2016). All the statistical analyses were performed in R 4.1.2 (www.r-project.org) with the TwoSampleMR .56 package.

## Results

We included 51 modifiable risk factors (exposure traits) in this Mendelian randomization. Eleven traits were classified into diets, sixteen were correlated to diet supplements or diet metabolites, thirteen were correlated to lifestyle factors, and eleven were cardiometabolic parameters. The number of SNPs correlated to each trait ranged from 7 to 437. Forty-five traits had a proportion of variance explained by SNPs larger than 1%. All the included traits and their SNPs passed the test of weak instrument test, with the F-statistic larger than 10 and the I2 GX over 90%. Table 1 shows the summary of the included traits and corresponding SNPs.

### Migraine without aura

**FIGURE 1:**
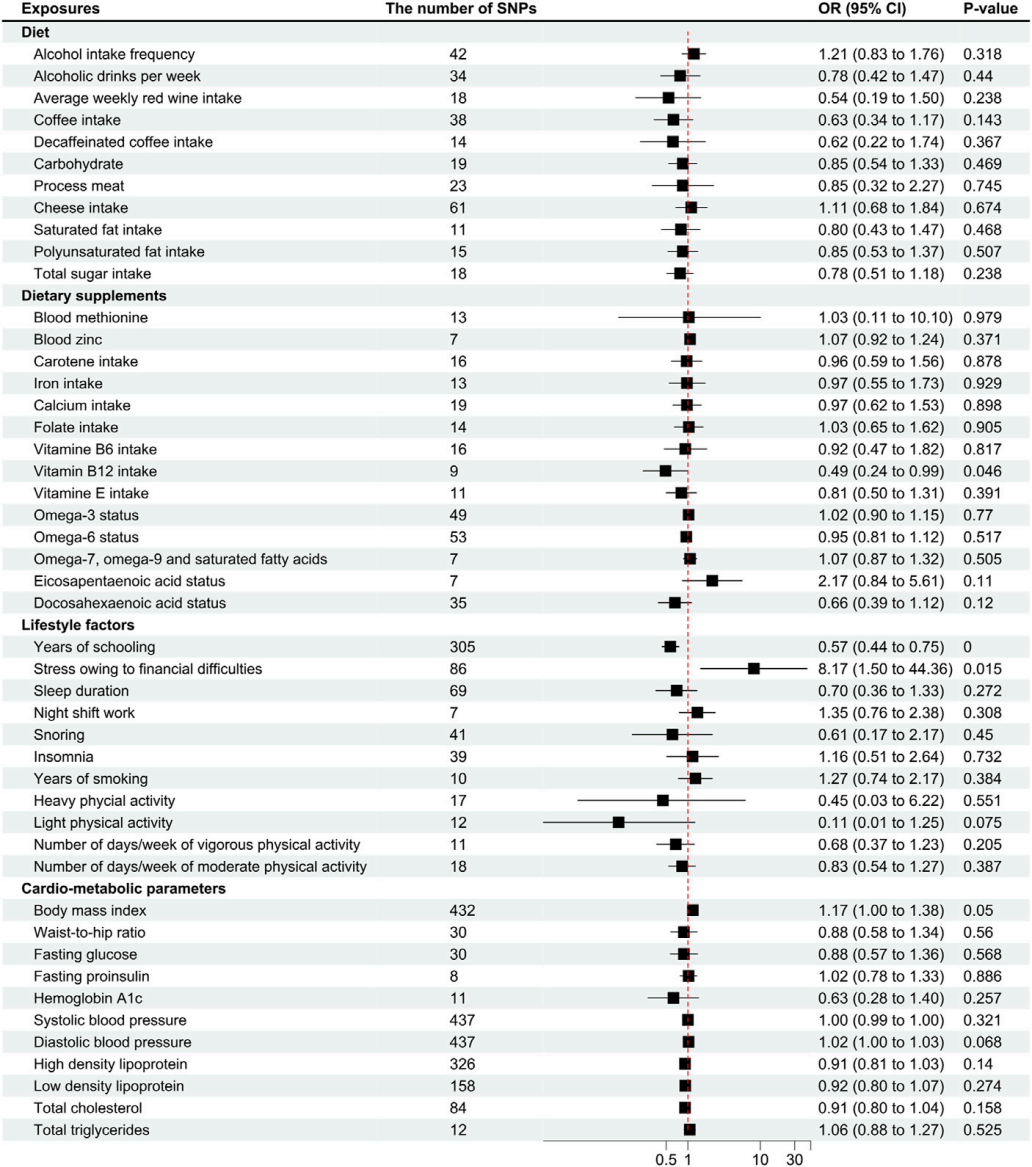
Odds ratios for associations between genetically predicted risk factors and migraine without aura. OR, odds ratio; SNP, single nucleotide polymorphisms. Annotation: We used the inverse-variance weighted method to calculate the odds ratios for individual SNPs. The odds ratio represents the odds ratio per genetically predicted SD unit increase in the risk factor. Take alcohol intake frequency for example, one SD unit increase in alcohol intake frequency was associated with 1.21 higher odds of migraine pathogenesis, which indicates that a higher frequency of alcohol intake was associated with a higher odds of migraine pathogenesis.

**FIGURE 2:**
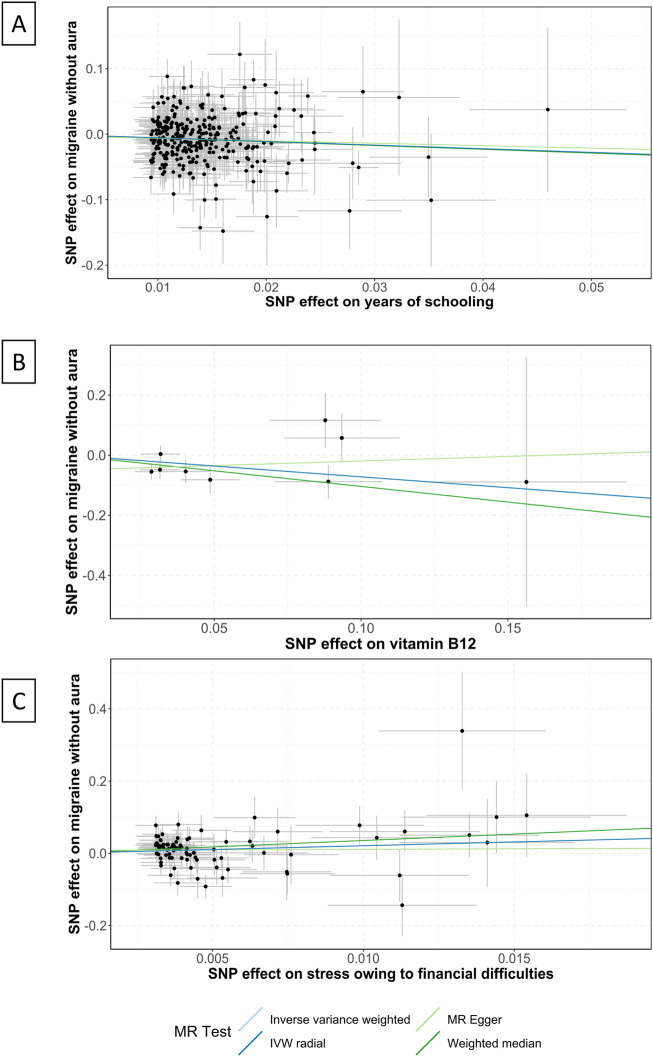
Scatter plots of the association between years of schooling, vitamin B12, and stress and migraine without aura. IVW, inverse variance weighted; MR, Mendelian randomization; SNP, single nucleotide polymorphisms. Annotation: Scatter plots of the migraine-SNP associations (*y*-axis) *versus* the exposures-SNP associations (*x*-axis) were shown, with vertical and horizontal lines showing 95% confidence intervals. The fitted curves among the dots refer to the differential statistical analysis methods. The MR analyses were performed with the inverse variance weighted method, the MR-Egger method, the weighted median method, and the IVW radial method. **(A)** shows the scatter plot for years of schooling, **(B)** shows the level of vitamin B12, and **(C)** shows the stress owing to financial difficulties.

Vitamin B12 intake was suggestive of a correlation with the pathogenesis of MwoA (OR .49 [95%CI .24 to .99], *p* = .046; Figure 1), and the weighted median analysis confirmed the finding (OR .35 [95%CI .15 to .85], *p* = .021; Supplementary eTable S2). As shown in Figure 2, the amount of vitamin B12 intake was inversely correlated to the incidence of migraine.

The factor years of schooling was found significantly correlated to the pathogenesis of MwoA (OR .57 [95%CI .44 to .75], *p* < .0001; Figure 1), and the weighted median analysis confirmed the finding (OR .56 [95%CI .39 to .81], *p* = .002; Supplementary eTable S2). An increase in the years of schooling was correlated with a lower incidence of migraine (Figure 2).

The stress owing to financial difficulties was suggestive of a correlation with the pathogenesis of migraine (OR 8.17 [95%CI 1.5 to 44.36], *p* = .015; Figure 1), and the weighted median analysis confirmed the finding (OR 34.85 [95%CI 3.31 to 366.42], *p* = .003; Supplementary eTable S2). Figure 2 shows that higher odds of stress were correlated to a higher incidence of migraine.

The anxiety disorder was also suggestive of a correlation with migraine (OR 1.92*10^9^ [95%CI 8.76 to 4.23*10^17^], *p* = .029), but the finding was not confirmed in the sensitivity analyses.

Other factors, like body mass index (OR 1.17 [95%CI one to 1.38], *p* = .05), light physical activity (OR .11 [95%CI .01 to 1.25], *p* = .075; Figure 1), and diastolic blood pressure (OR 1.02 [95%CI one to 1.03], *p* = .068), were close to the suggestive boundary of a correlation with migraine pathogenesis. In addition, an MR-Egger analysis suggested a causal effect of total cholesterol level on migraine pathogenesis (OR .77 [95%CI .63 to .96], *p* = .068; Supplementary eTable S3), although it was not confirmed in the IVW analysis.

The other traits had no significant or causal effect on migraine without aura in all the analyses (Figures 1, 2, and Supplementary eTable S2–S4). Heterogeneity was not found in the analyses (Supplementary eTable S5).

### Migraine with aura

**FIGURE 3:**
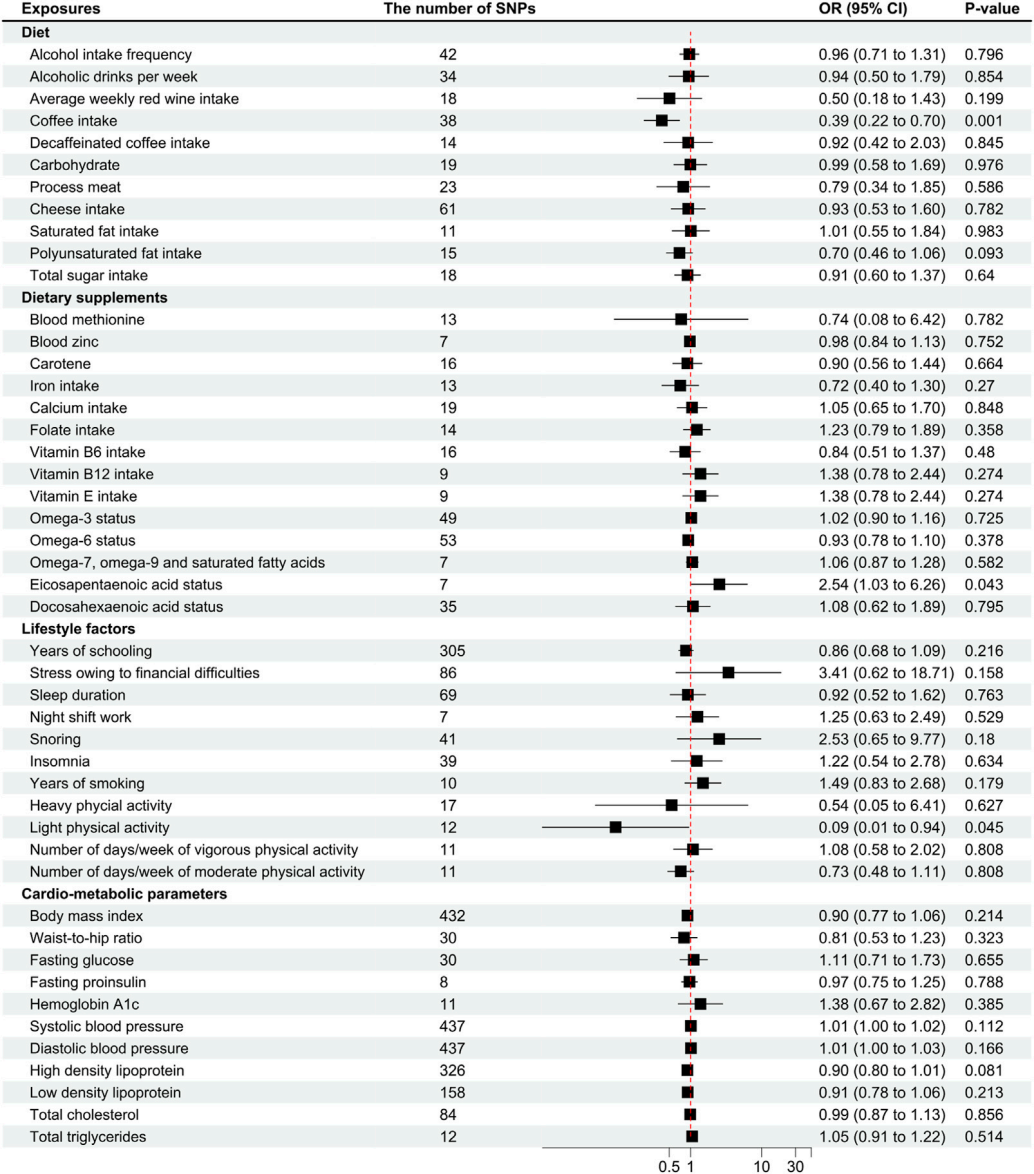
Odds ratios for associations between genetically predicted risk factors and migraine with aura. OR, odds ratio; SNP, single nucleotide polymorphisms. Annotation: We used the inverse-variance weighted method to calculate the odds ratios for individual SNPs. The odds ratio represents the odds ratio per genetically predicted SD unit increase in the risk factor.

**FIGURE 4:**
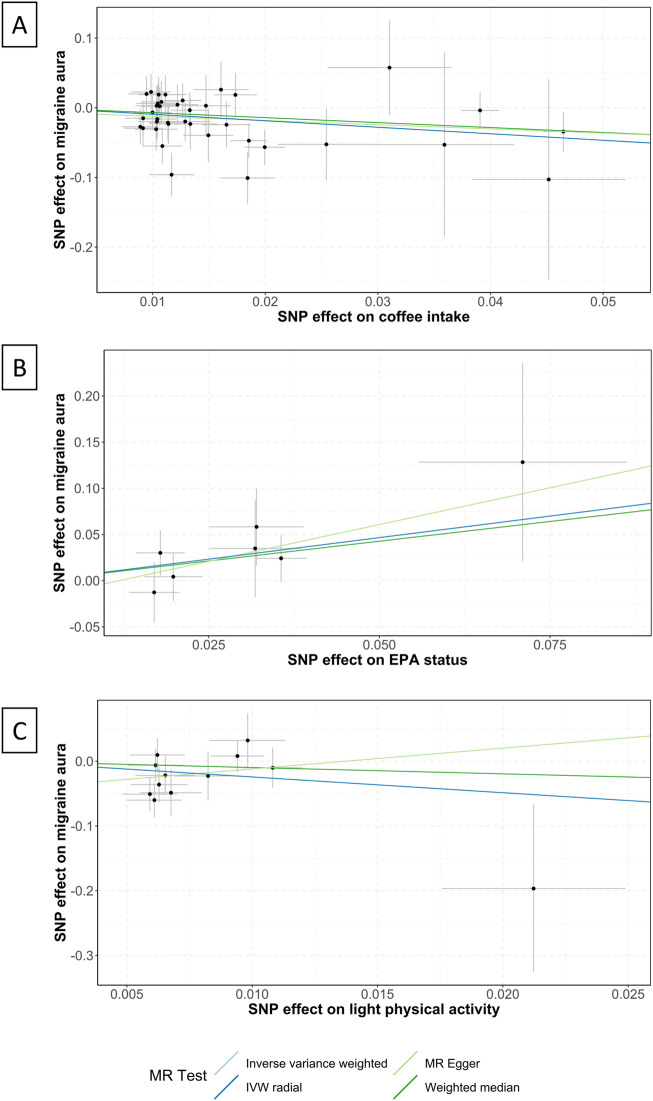
Scatter plots of the association between coffee intake, EPA status, light physical activity and migraine with aura. EPA, Eicosapentaenoic acid; IVW, inverse variance weighted; MR, Mendelian randomization; SNP, single nucleotide polymorphisms. Annotation: Scatter plots of the migraine-SNP associations (*y*-axis) *versus* the exposures-SNP associations (*x*-axis) were shown, with vertical and horizontal lines showing 95% confidence intervals. The fitted curves among the dots refer to the differential statistical analysis methods. The MR analyses were performed with the inverse variance weighted method, the MR-Egger method, the weighted median method, and the IVW radial method. **(A)** shows the scatter plot of coffee intake, **(B)** shows EPA status, and **(C)** shows light physical activity.

Coffee intake was found suggestively correlated with the pathogenesis of MwA (OR .39 [95%CI .22 to .7], *p* = .001; Figure 3), but this finding was not supported by other sensitivity analyses (Supplementary eTable S6–S8). Coffee intake was inversely correlated with MwA, higher odds of genetically predicted coffee intake correlate with lower odds of MwA pathogenesis (Figure 4).

Eicosapentaenoic acid status was suggestive of a correlation with MwA (OR 2.54 [95%CI 1.03 to 6.26], *p* = .043; Figure 3), but this finding was not confirmed in subsequent sensitivity analyses (Supplementary eTable S6–S8). A higher level of Eicosapentaenoic acid status was indicative of higher odds of MwA pathogenesis (Figure 4).

Light physical activity, instead of heavy physical activity, was suggested to correlate with MwA pathogenesis (OR .09 [95%CI .01 to .94], *p* = .046; Figure 3), although this finding was not confirmed in other sensitivity analyses. A higher frequency of light physical activity was correlated with lower odds of MwA pathogenesis (Figure 4).

Fasting glucose and HbA1c, as suggested by the weighted median analysis, were correlated with MwA pathogenesis (fasting glucose, OR 2.08 [95%CI 1.14 to 3.79], *p* = .016; HbA1c, OR 2.53 [95%CI 1.11 to 5.74], *p* = .02; Supplementary eTable S6). Higher levels of fasting glucose and HbA1c caused higher odds of MwA. The MR-Egger analysis showed that sleep duration correlated with MwA pathogenesis (OR 10.49 [95%CI 1.09 to 101.26], *p* = .046; Supplementary eTable S7).

Polyunsaturated fat, zinc status, vitamin D, years of schooling, depression, anxiety disorders, snoring, body mass index, and DBP were possibly correlated with MwA pathogenesis, which had *p*-values between .05 and .1 (Figure 3). The other risk factors had no significant or suggestive correlation with MwA pathogenesis (Figure 3, Supplementary eTable S6–S8). Heterogeneity was not found in the analyses (Supplementary eTable S9).

## Discussion

We found that, in the Mendelian randomization analysis, genetically predicted years of schooling, intake of vitamin B12, and stress were causally correlated with MwoA. More years of schooling, a larger amount of intake of vitamin B12, and lower odds of stress were correlated with lower incidence of MwoA. We also found that genetically predicted coffee intake, EPA status, and light physical activity were causally correlated with MwA. A larger amount of coffee intake and higher odds of light physical activity were correlated with a lower risk of MwA, and a higher level of EPA was correlated with a higher risk of MwA. In the aforementioned correlations, only the years of schooling were statistically significant, the others were suggestive.

Our study adopted an MR design, which minimized confounding bias and reverse causation. Before performing the MR analysis, we implemented a comprehensive search for previously reported modifiable risk factors for migraine, so our study covered a wide range of risk factors for migraine, which is meaningful for developing multidisciplinary strategies for migraine prevention. In addition, we selected exposures mostly from United Kingdom Biobank and outcome data from the FinnGen study, which, to a certain extent, avoided the sample overlap and reduced bias from the overlap.

Years of schooling were found to be significantly associated with MwoA, and more years of schooling correlated with lower rates of MwoA. Previous studies reported that higher prevalence rates of migraine were correlated with lower education levels (Wang et al., 2016; Brusa et al., 2017; Brusa et al., 2019). We assumed that individuals with more years of education might be more voluntarily looking for access to migraine prophylaxis (Spadea et al., 2019) and acquiring a guideline-recommended strategy for migraine to avoid medication overuse headaches (Radtke and Neuhauser, 2012). This finding informed that headache education for a broader population is essential, and a recent randomized controlled trial showed that headache education could have a 2-day decrease in monthly headache days (Wells et al., 2021).

We found that a higher frequency of light physical activity was suggestively associated with lower odds of MwA and possibly correlative with MwoA (*p* = .075). These findings indicated a protective effect of light physical activity on migraine. Previous reviews concluded a beneficial effect of regular exercise on migraine prevention, but whether a higher exercise intensity leads to better prevention is unknown (Krøll et al., 2017; Amin et al., 2018; Lemmens et al., 2019; Barber and Pace, 2020). Our study demonstrated that light physical activity was more likely to be beneficial than heavy activity. The mechanism of exercise for migraine prophylaxis might include increased levels of beta-endorphin, endocannabinoid, and brain-derived neurotrophic factor in plasma after exercise (Amin et al., 2018).

Vitamin B12 and EPA were associated with the pathogenesis of migraine. More vitamin B12 intake caused lower odds of migraine, while higher EPA levels induced higher odds of migraine. Previous studies indicated that the high level of homocysteine was the cause of migraine, and B vitamins, especially vitamin B12, catalyzed homocysteine and therefore were beneficial for migraine prevention (Shaik et al., 2014; Urits et al., 2020). In addition, a recent systematic review showed that patients with migraine presented with a higher level of homocysteine and a lower level of folate, which also confirmed a relationship between B vitamins and migraine pathogenesis (Liampas et al., 2020). The mechanism of how EPA affects migraine is still unknown, which might warrant further investigation.

We found that more coffee intake was associated with a lower rate of MwoA, which might be the effect of caffeine. Caffeine, as a cure for acute migraine attacks, is assumed to antagonize adenosine and therefore decrease the production of NO, which is believed to be a major trigger of migraine attacks. Although migraine headaches might become chronic owing to caffeine withdrawal, it is reported that only 5%–30% of the patients with migraine had higher attack frequencies after caffeine withdrawal, and the migraine attack frequency might gradually decrease when patients keep a stable use of caffeine (<200 mg/day) (Nowaczewska et al., 2020).

Migraine was comorbid with several psychiatric disorders. In our study, we found that stress had a suggestive causal effect on migraine, and anxiety and depression were possibly associated with migraine pathogenesis. These findings were correlated with previous studies, and the effectiveness of amitriptyline for migraine confirmed the causal effect of the psychiatric disorders (Couch, 2011). The mechanism of stress, anxiety, and depression causing migraine mainly focused on the abnormal function, structure, and connectivity of brain regions—anterior cingulate cortex, anterior insula, prefrontal cortex, hippocampus, and amygdala (Minen et al., 2016). One recent study demonstrated that occipital bending was associated with migraine with visual aura, which in part explains psychiatric disorders in patients with MwA (Özkan and Gürsoy-Özdemir, 2021). Another study that found frontotemporal hypometabolism and increased frontal cortical thickness in patients with chronic migraine might explain some cognitive and behavioral pain-processing and sensory integration alterations in patients with migraine (Torres-Ferrus et al., 2021).

Taking prophylactic medications for migraine and the difference between episodic migraine and chronic migraine in disease nature might affect the results of the study. Owing to the lack of information on these points, we were unable to clarify whether these two factors are important confounding factors.

Our study still had several limitations. First, we adopted summary-level data in the analysis, which might ignore important covariates that influence the study result. Second, we screened potentially modifiable risk factors reported in previously published studies and examined these risk factors. Those that were unreported might also affect migraine pathogenesis. Third, genetically predicted exposures have a lifetime effect on migraine, so the effect size of these exposures might be exaggerated. Fourth, the GWAS study data were from the European population, and the results of this Mendelian randomization might only be generalized to the European population. Fifth, the risk factors that had an insignificant correlation with migraine in this study might be the consequence of low statistical power.

In conclusion, we found that years of schooling were significantly associated with migraine pathogenesis, and stress, light physical activity, vitamin B12 intake, coffee intake, and EPA status were suggestive of correlations. Comprehensive interventions might be developed based on modifying these factors for migraine prophylaxis.

## Data Availability

Publicly available datasets were analyzed in this study. This data can be found here: pan.baidu.com.

## Supplementary material

Supplementary eTable 1 Reference articles reporting risk factors of migraine pathogenesis

Supplementary eTable 2 Weighted median analysis for migraine without aura (MwoA).

Supplementary eTable 3 MR-Egger analysis for migraine without aura (MwoA).

Supplementary eTable 4 IVW radial analysis for migraine without aura (MwoA).

Supplementary eTable 5 Heterogeneity test (MwoA).

Supplementary eTable 6 Weighted median analysis of risk factors of MwA.

Supplementary eTable 7 MR-Egger analysis of risk factors of MwA.

Supplementary eTable 8 IVW Radial analysis (MwA).

Supplementary eTable 9 Heterogeneity test (MwA).
