# Supplementary material for: Modifiable factors for migraine prophylaxis: A mendelian randomization analysis
Source: Front Pharmacol. 2023 Jan 12;14:1010996. doi: 10.3389/fphar.2023.1010996 (PMC9878312; doi:10.3389/fphar.2023.1010996)
Supplement: Supplementary file 1 [file DataSheet1.pdf]

Modifiable risk factors for the prevention of migraine: a mendelian randomization analysis

Contents

eTable 1. Reference articles reporting risk factors of migraine pathogenesis 2

eTable 2. Weighted median analysis for migraine without aura (MwoA) 4

eTable 3. MR-Egger analysis for migraine without aura (MwoA) 6

eTable 4. IVW radial analysis for migraine without aura (MwoA) 8

eTable 5. Heterogeneity test (MwoA) 10

eTable 6. Weighted median analysis of risk factors of MwA 13

eTable 7. MR-Egger analysis of risk factors of MwA 15

eTable 8. IVW Radial analysis (MwA) 17

eTable 9. Heterogeneity test (MwA) 19

eTable 1. Reference articles reporting risk factors of migraine pathogenesis

No.	Reference articles
1	Amin FM, Aristeidou S, Baraldi C, et al. The association between migraine and physical exercise. J Headache Pain. 2018;19(1):83. doi:10.1186/s10194-018-0902-y
2	Ashina M, Buse DC, Ashina H, et al. Migraine: integrated approaches to clinical management and emerging treatments. Lancet. 2021;397(10283):1505-1518. doi:10.1016/S0140-6736(20)32342-4
3	Ashina M, Katsarava Z, Do TP, et al. Migraine: epidemiology and systems of care. Lancet. 2021;397(10283):1485-1495. doi:10.1016/S0140-6736(20)32160-7
4	Ashina M, Terwindt GM, Al-Karagholi MAM, et al. Migraine: disease characterisation, biomarkers, and precision medicine. Lancet. 2021;397(10283):1496-1504. doi:10.1016/S0140-6736(20)32162-0
5	Assarzadegan F, Hosseinpanahi SP, Hesami O, Mansouri B, Lima BS. Frequency of dyslipidemia in migraineurs in comparison to control group. J Family Med Prim Care. 2019;8(3):950-954. doi:10.4103/jfmpc.jfmpc_9_19
6	Barber M, Pace A. Exercise and Migraine Prevention: a Review of the Literature. Curr Pain Headache Rep. 2020;24(8):39. doi:10.1007/s11916-020-00868-6
7	Buse DC, Greisman JD, Baigi K, Lipton RB. Migraine Progression: A Systematic Review. Headache. 2019;59(3):306-338. doi:10.1111/head.13459
8	Dasari VR, Clark AJ, Boorigie ME, Gerson T, Connelly MA, Bickel JL. The Influence of Lifestyle Factors on the Burden of Pediatric Migraine. J Pediatr Nurs. 2021;57:79-83. doi:10.1016/j.pedn.2020.12.003
9	Gazerani P. Migraine and Diet. Nutrients. 2020;12(6). doi:10.3390/nu12061658
10	Goadsby PJ, Holland PR, Martins-Oliveira M, Hoffmann J, Schankin C, Akerman S. Pathophysiology of Migraine: A Disorder of Sensory Processing. Physiol Rev. 2017;97(2):553-622. doi:10.1152/physrev.00034.2015
11	Hagen K, Åsberg AN, Stovner L, et al. Lifestyle factors and risk of migraine and tension-type headache. Follow-up data from the Nord-Trøndelag Health Surveys 1995-1997 and 2006-2008. Cephalalgia. 2018;38(13):1919-1926. doi:10.1177/0333102418764888
12	Krøll LS, Hammarlund CS, Westergaard ML, et al. Level of physical activity, well-being, stress and self-rated health in persons with migraine and co-existing tension-type headache and neck pain. J Headache Pain. 2017;18(1):46. doi:10.1186/s10194-017-0753-y
13	Lemmens J, De Pauw J, Van Soom T, et al. The effect of aerobic exercise on the number of migraine days, duration and pain intensity in migraine: a systematic literature review and meta-analysis. J Headache Pain. 2019;20(1):16. doi:10.1186/s10194-019-0961-8

14 Leso V, Gervetti P, Mauro S, Macrini MC, Ercolano ML, Iavicoli I. Shift work and migraine: A systematic review. J Occup Health. 2020;62(1):e12116. doi:10.1002/1348-9585.12116

15 Liampas I, Mylonas KS, Brotis A, et al. Serum lipid abnormalities in migraine: A meta-analysis of observational studies. Headache. 2021;61(1):44-59. doi:10.1111/head.14039

16 Liampas I, Siokas V, Mentis AFA, et al. Serum Homocysteine, Pyridoxine, Folate, and Vitamin B12 Levels in Migraine: Systematic Review and Meta-Analysis. Headache. 2020;60(8):1508-1534. doi:10.1111/head.13892

17 Marmura MJ. Triggers, Protectors, and Predictors in Episodic Migraine. Curr Pain Headache Rep. 2018;22(12):81. doi:10.1007/s11916-018-0734-0

18 May A, Schulte LH. Chronic migraine: risk factors, mechanisms and treatment. Nat Rev Neurol. 2016;12(8):455-464. doi:10.1038/nrneurol.2016.93

19 Minen MT, Begasse De Dhaem O, Kroon Van Diest A, et al. Migraine and its psychiatric comorbidities. J Neurol Neurosurg Psychiatry. 2016;87(7):741-749. doi:10.1136/jnnp-2015-312233

20 Nazari F, Safavi M, Mahmudi M. Migraine and its relation with lifestyle in women. Pain Pract. 2010;10(3):228-234. doi:10.1111/j.1533-2500.2009.00343.x

21 Nowaczewska M, Wiciński M, Kaźmierczak W. The Ambiguous Role of Caffeine in Migraine Headache: From Trigger to Treatment. Nutrients. 2020;12(8):E2259. doi:10.3390/nu12082259

22 Puledda F, Messina R, Goadsby PJ. An update on migraine: current understanding and future directions. Journal of Neurology. 2017;264(9):2031. doi:10.1007/s00415-017-8434-y

23 Robblee J, Starling AJ. SEEDS for success: Lifestyle management in migraine. CCJM. 2019;86(11):741-749. doi:10.3949/ccjm.86a.19009

24 Shaik MM, Tan HL, Kamal MA, Gan SH. Do folate, vitamins B_6 and B_{12} play a role in the pathogenesis of migraine? The role of pharmacoepigenomics. CNS Neurol Disord Drug Targets. 2014;13(5):828-835. doi:10.2174/18715273113129990112

25 Stubberud A, Buse DC, Kristoffersen ES, Linde M, Tronvik E. Is there a causal relationship between stress and migraine? Current evidence and implications for management. J Headache Pain. 2021;22(1):155. doi:10.1186/s10194-021-01369-6

26 Urits I, Yilmaz M, Bahrun E, et al. Utilization of B12 for the treatment of chronic migraine. Best Pract Res Clin Anaesthesiol. 2020;34(3):479-491. doi:10.1016/j.bpa.2020.07.009

eTable 2. Weighted median analysis for migraine without aura (MwoA)

Exposure	No. of SNP	Beta	se	P-value	OR	95%CI lower bound	95%CI upper bound
Alcohol intake frequency	42	0.044545	0.258606	0.863242	1.045551689	0.6298	1.7357
Alcoholic drinks per week	34	-0.44603	0.466397	0.338899	0.640161424	0.2566	1.597
Average weekly red wine intake	18	-0.24116	0.694223	0.728308	0.785718154	0.2015	3.0634
Coffee intake	38	-0.35536	0.430641	0.40926	0.700918653	0.3014	1.6302
Decaffeinated coffee intake	14	-0.3683	0.630371	0.559046	0.691909254	0.2011	2.3803
Carbohydrate	19	0.10096	0.306928	0.742204	1.106232135	0.6062	2.0189
Process meat	23	0.066917	0.619435	0.913972	1.069207175	0.3175	3.6003
Cheese intake	61	-0.19326	0.349818	0.58064	0.82427013	0.4152	1.6362
Saturated fat intake	11	0.017369	0.423576	0.967292	1.017520517	0.4436	2.334
Polyunsaturated fat intake	15	-0.49051	0.319181	0.124352	0.612316636	0.3276	1.1446
Total sugar intake	18	-0.2146	0.296171	0.468717	0.806867553	0.4515	1.4418
Blood methionine	13	-0.77815	1.590605	0.624689	0.459255185	0.0203	10.375
Blood selenium	12	2.034245	3.527401	0.564144	7.646473848	0.0076	7692.1
Blood zinc	7	0.147272	0.079867	0.065188	1.158669037	0.9908	1.355
Vitamin D intake	14	7.666355	5.463248	0.160539	2135.283721	0.0478	1E+08
Carotene intake	16	0.139665	0.329805	0.671948	1.149888005	0.6024	2.1948
Iron intake	13	-0.14519	0.393474	0.712125	0.864854984	0.4	1.8701
Calcium intake	19	-0.06902	0.324528	0.831574	0.933306299	0.4941	1.7631
Folate intake	14	0.124083	0.333338	0.709711	1.132110062	0.589	2.1759
Vitamin B6 intake	16	0.080377	0.396694	0.839434	1.08369572	0.498	2.3582
Vitamin B12 intake	9	-1.03943	0.448706	0.020531	0.353655544	0.1468	0.8522
Vitamin E intake	11	0.228044	0.315396	0.469656	1.256140404	0.677	2.3308
Omega-3 status	49	0.036071	0.073731	0.624687	1.036728958	0.8972	1.1979
Omega-6 status	53	-0.03556	0.131617	0.787046	0.965068451	0.7456	1.2491
Omega-7, omega-9, and saturated fatty acids	7	0.091391	0.138686	0.509912	1.09569703	0.8349	1.4379
Eicosapentaenoic acid status	7	0.360106	0.602642	0.550143	1.433481842	0.44	4.6706
Docosahexaenoic acid status	35	-0.69262	0.389912	0.075677	0.500265227	0.233	1.0742
Years of schooling	305	-0.57696	0.189367	0.002313	0.561600388	0.3875	0.814
Stress owing to financial difficulties	86	3.551321	1.200246	0.003088	34.85932046	3.3163	366.43
Anxiety disorder	17	22.17949	13.36347	0.096973	4289746330	0.0181	######
Depression	31	-0.24121	2.962999	0.935118	0.785675752	0.0024	261.46
Sleep duration	69	-0.12025	0.465819	0.796292	0.886698037	0.3558	2.2095

Night shift work	7	0.11231	0.37809	0.766431	1.118859824	0.5333	2.3475
Snoring	41	-0.3849	0.938362	0.68167	0.680517137	0.1082	4.2814
Insomnia	39	-0.73802	0.619344	0.233412	0.4780598	0.142	1.6095
Years of smoking	10	0.143755	0.34503	0.676939	1.154600696	0.5871	2.2705
Heavy physical activity	17	-0.24383	1.7571	0.889631	0.783617522	0.025	24.535
Light physical activity	12	-2.5827	1.708111	0.130528	0.075569645	0.0027	2.1494
Number of days/weeks of vigorous physical activity	11	-0.23922	0.400391	0.550198	0.787242051	0.3592	1.7256
Number of days/weeks of moderate physical activity	18	0.040923	0.282823	0.884951	1.041772193	0.5985	1.8135
Body mass index	432	-0.05495	0.142448	0.699689	0.946534349	0.7159	1.2514
Waist-to-hip ratio	30	-0.35577	0.289522	0.21914	0.700634214	0.3972	1.2358
Fasting glucose	30	-0.22955	0.320554	0.473921	0.794888816	0.4241	1.4899
Fasting proinsulin	8	0.058108	0.185029	0.753485	1.059829565	0.7375	1.5231
Hemoglobin A1c	11	-0.49968	0.403394	0.21546	0.606724604	0.2752	1.3377
Systolic blood pressure	437	-0.01264	0.007665	0.099035	0.987435896	0.9727	1.0024
Diastolic blood pressure	437	0.001963	0.012957	0.879604	1.001964555	0.9768	1.0277
High density lipoprotein	326	-0.18255	0.111965	0.103003	0.833138878	0.669	1.0376
Low density lipoprotein	158	-0.11745	0.118916	0.323326	0.88918742	0.7043	1.1226
Total cholesterol	84	-0.18738	0.103232	0.069495	0.829124653	0.6772	1.0151
Total triglycerides	12	0.077631	0.11593	0.503087	1.080724287	0.8611	1.3564

eTable 3. MR-Egger analysis for migraine without aura (MwoA)

Exposure	No. of SNP	Beta	se	P-value	OR	95%CI lower bound	95%CI upper bound
Alcohol intake frequency	42	-0.2859	0.546947	0.604052	0.751337	0.257196	2.194855
Alcoholic drinks per week	34	0.115252	0.742284	0.877586	1.122157	0.261944	4.807271
Average weekly red wine intake	18	1.742635	2.526081	0.500168	5.712375	0.040418	807.3475
Coffee intake	38	-0.36647	0.637051	0.568691	0.693175	0.198872	2.416081
Decaffeinated coffee intake	14	-0.61879	0.956572	0.529884	0.538593	0.082606	3.511653
Carbohydrate	19	-0.53467	0.623568	0.403128	0.585861	0.172585	1.988781
Process meat	23	2.182983	2.530413	0.398049	8.872733	0.062248	1264.703
Cheese intake	61	0.430733	1.08542	0.69292	1.538385	0.183289	12.91199
Saturated fat intake	11	-1.88481	0.936701	0.075064	0.151858	0.024216	0.952301
Polyunsaturated fat intake	15	-0.25772	0.45752	0.582814	0.772809	0.315227	1.894615
Total sugar intake	18	-0.48933	0.451782	0.294817	0.61304	0.252885	1.48612
Blood methionine	13	6.468834	3.81334	0.117897	644.7316	0.36593	1135952
Blood selenium	12	24.15379	51.00037	0.645955	3.09E+10	1.2E-33	7.98E+53
Blood zinc	7	0.235158	0.308028	0.479659	1.265109	0.691718	2.313805
Vitamin D intake	14	26.20825	18.1969	0.175371	2.41E+11	7.81E-05	7.44E+26
Carotene intake	16	0.090179	0.528443	0.86694	1.094371	0.388459	3.083075
Iron intake	13	-0.28143	0.765325	0.720057	0.754706	0.168391	3.38248
Calcium intake	19	-1.4376	0.890781	0.124965	0.237498	0.041439	1.361157
Folate intake	14	0.13362	0.438175	0.765631	1.142959	0.484226	2.697818
Vitamin B6 intake	16	-0.77846	0.796924	0.345231	0.459113	0.096286	2.189146
Vitamin B12 intake	9	0.304947	0.802174	0.715106	1.356552	0.281587	6.535225
Vitamin E intake	11	0.260825	0.484371	0.603305	1.298001	0.502308	3.354133
Omega-3 status	49	0.074332	0.085638	0.389816	1.077165	0.910721	1.274028
Omega-6 status	53	-0.12677	0.167378	0.452289	0.880933	0.634553	1.222975
Omega-7, omega-9, and saturated fatty acids	7	0.596644	0.421332	0.215919	1.816013	0.795195	4.147288
Eicosapentaenoic acid status	7	-0.304	1.378504	0.834179	0.737858	0.049496	10.99965
Docosahexaenoic acid status	35	-0.44025	0.67059	0.516044	0.643873	0.172975	2.396725
Years of schooling	305	-0.37612	0.531347	0.479579	0.686522	0.242305	1.945117
Stress owing to financial difficulties	86	0.290065	2.606228	0.911646	1.336514	0.008082	221.0243
Anxiety disorder	17	4.221181	21.10368	0.844151	68.11386	7.4E-17	6.27E+19
Depression	31	-4.10716	6.299766	0.519565	0.016454	7.14E-08	3790.988
Sleep duration	69	1.220952	1.300847	0.351317	3.390412	0.264819	43.40665

Night shift work	7	0.442115	0.887633	0.639556	1.555995	0.273174	8.862914
Snoring	41	-2.73391	3.408674	0.427387	0.064965	8.15E-05	51.78435
Insomnia	39	-1.4231	1.20971	0.246949	0.240966	0.022502	2.580364
Years of smoking	10	-0.13078	0.638415	0.84281	0.877415	0.251059	3.066441
Heavy physical activity	17	-0.92317	8.710355	0.916999	0.397259	1.53E-08	10315194
Light physical activity	12	-1.42637	5.14343	0.787184	0.24018	1.01E-05	5737.368
Number of days/weeks of vigorous physical activity	11	-0.74284	2.416045	0.765487	0.475759	0.004176	54.1958
Number of days/weeks of moderate physical activity	18	-1.18113	1.342258	0.39191	0.306933	0.022105	4.261842
Body mass index	432	-0.27649	0.221558	0.212741	0.758444	0.49128	1.170895
Waist-to-hip ratio	30	0.128864	0.932648	0.891095	1.137536	0.182843	7.077034
Fasting glucose	30	-0.71996	0.469365	0.136275	0.48677	0.193996	1.221394
Fasting proinsulin	8	0.202068	0.342475	0.576705	1.223931	0.625513	2.394848
Hemoglobin A1c	11	-0.02044	1.089306	0.98544	0.979769	0.115848	8.286286
Systolic blood pressure	437	0.00013	0.012479	0.991708	1.00013	0.975964	1.024894
Diastolic blood pressure	437	-0.00157	0.020869	0.940201	0.998435	0.95842	1.04012
High density lipoprotein	326	-0.13209	0.09552	0.167654	0.876259	0.726648	1.056675
Low density lipoprotein	158	-0.13872	0.108029	0.201017	0.870473	0.704368	1.075751
Total cholesterol	84	-0.25364	0.10744	0.020614	0.775975	0.628627	0.957861
Total triglycerides	12	-0.08829	0.2718	0.751987	0.915491	0.537394	1.559607

eTable 4. IVW radial analysis for migraine without aura (MwoA)

Exposure	No. of SNP	Beta	se	P-value	OR	95%CI lower bound	95%CI upper bound
Alcohol intake frequency	42	0.191017	0.191265	0.317939	1.21048	0.83205	1.761027
Alcoholic drinks per week	34	-0.24864	0.289216	0.38995	0.77986	0.442416	1.374681
Average weekly red wine intake	18	-0.61792	0.523726	0.238059	0.539065	0.193124	1.504686
Coffee intake	38	-0.4576	0.312502	0.143113	0.632803	0.342974	1.167551
Decaffeinated coffee intake	14	-0.47212	0.523705	0.367324	0.62368	0.223447	1.740797
Carbohydrate	19	-0.16751	0.172397	0.33121	0.845764	0.603257	1.185759
Process meat	23	-0.16364	0.502352	0.74462	0.84905	0.317192	2.272711
Cheese intake	61	0.107281	0.255189	0.674193	1.113248	0.675104	1.835748
Saturated fat intake	11	-0.22738	0.283116	0.421901	0.796619	0.457359	1.387535
Polyunsaturated fat intake	15	-0.16244	0.24531	0.507864	0.85007	0.525584	1.374888
Total sugar intake	18	-0.2515	0.182625	0.168462	0.77763	0.543651	1.112312
Blood methionine	13	0.03024	1.152497	0.979067	1.030702	0.107673	9.866401
Blood selenium	12	1.783807	2.166252	0.41025	5.952472	0.08526	415.5738
Blood zinc	7	0.067822	0.075962	0.371945	1.070174	0.922135	1.24198
Vitamin D intake	14	6.042302	4.072644	0.137907	420.8606	0.143692	1232659
Carotene intake	16	-0.03775	0.178502	0.832523	0.962956	0.678676	1.366315
Iron intake	13	-0.02589	0.245849	0.916143	0.974446	0.601848	1.577715
Calcium intake	19	-0.02995	0.226578	0.894835	0.970493	0.622479	1.513073
Folate intake	14	0.027732	0.207318	0.89359	1.02812	0.684811	1.543535
Vitamin B6 intake	16	-0.08051	0.347014	0.816535	0.922647	0.46736	1.821461
Vitamin B12 intake	9	-0.72025	0.362992	0.047232	0.486629	0.238898	0.99125
Vitamin E intake	11	-0.21128	0.231941	0.362339	0.809549	0.51382	1.275483
Omega-3 status	49	0.017978	0.061438	0.76981	1.018141	0.902633	1.14843
Omega-6 status	53	-0.05382	0.081164	0.507235	0.947599	0.808232	1.110997
Omega-7, omega-9, and saturated fatty acids	7	0.069528	0.09465	0.462598	1.072002	0.890487	1.290517
Eicosapentaenoic acid status	7	0.773604	0.38599	0.045048	2.167564	1.017211	4.618841
Docosahexaenoic acid status	35	-0.41869	0.269186	0.119848	0.657905	0.388175	1.115061
Years of schooling	305	-0.55461	0.135686	4.36E-05	0.574298	0.440189	0.749265
Stress owing to financial difficulties	86	2.099185	0.863611	0.015069	8.159514	1.501564	44.33888
Anxiety disorder	17	21.45353	9.837375	0.029197	2.08E+09	8.778219	4.91E+17
Depression	31	1.433766	2.256172	0.525112	4.194464	0.050371	349.2762
Sleep duration	69	-0.36231	0.330022	0.272279	0.696068	0.364529	1.329143
Night shift work	7	0.296614	0.181587	0.102373	1.345296	0.942428	1.920381
Snoring	41	-0.48649	0.643379	0.449562	0.614782	0.174207	2.169582
Insomnia	39	0.143205	0.421436	0.734006	1.153966	0.505195	2.635887
Years of smoking	10	0.237871	0.235878	0.313239	1.268545	0.798956	2.014139

Heavy physical activity	17	-0.79845	1.339955	0.551259	0.450028	0.032557	6.220605
Light physical activity	12	-2.18117	1.225756	0.075166	0.112909	0.010218	1.247714
Number of days/weeks of vigorous physical activity	11	-0.38596	0.274156	0.159191	0.6798	0.397205	1.16345
Number of days/weeks of moderate physical activity	18	-0.18673	0.182506	0.306238	0.829668	0.580166	1.186468
Body mass index	432	0.160367	0.081912	0.050254	1.173942	0.999819	1.378389
Waist-to-hip ratio	30	-0.12356	0.181775	0.496676	0.883771	0.618884	1.262029
Fasting glucose	30	-0.12794	0.223794	0.567547	0.87991	0.567467	1.364381
Fasting proinsulin	8	0.019742	0.137042	0.885453	1.019938	0.77969	1.334216
Hemoglobin A1c	11	-0.4647	0.409957	0.256987	0.628322	0.281332	1.403281
Systolic blood pressure	437	-0.00492	0.004955	0.320817	0.995093	0.985475	1.004804
Diastolic blood pressure	437	0.01569	0.008602	0.068146	1.015814	0.998831	1.033086
High density lipoprotein	326	-0.09285	0.062668	0.138458	0.911333	0.805995	1.030437
Low density lipoprotein	158	-0.08061	0.073631	0.273621	0.922555	0.798576	1.065781
Total cholesterol	84	-0.09468	0.067007	0.157647	0.909662	0.797706	1.037331
Total triglycerides	12	0.059238	0.093157	0.524848	1.061027	0.883954	1.273573

eTable 5. Heterogeneity test (MwoA)

Exposure	Method	Q-value	Q_df	P-value
Alcohol intake frequency	Inverse variance weighted	54.51262	41	0.076951
Alcoholic drinks per week	Inverse variance weighted	26.66417	33	0.774184
Average weekly red wine intake	Inverse variance weighted	19.39142	17	0.306533
Coffee intake	Inverse variance weighted	38.29645	37	0.410461
Decaffeinated coffee intake	Inverse variance weighted	22.82021	13	0.043885
Carbohydrate	Inverse variance weighted	9.985644	18	0.932374
Process meat	Inverse variance weighted	30.77157	22	0.100885
Cheese intake	Inverse variance weighted	67.94857	60	0.224797
Saturated fat intake	Inverse variance weighted	8.14712	10	0.614469
Polyunsaturated fat intake	Inverse variance weighted	16.29944	14	0.295432
Total sugar intake	Inverse variance weighted	12.50989	17	0.768328
Blood methionine	Inverse variance weighted	11.7526	12	0.465747
Blood selenium	Inverse variance weighted	8.131778	11	0.701453
Blood zinc	Inverse variance weighted	8.635173	6	0.195159
Vitamin D intake	Inverse variance weighted	15.09206	13	0.301641
Carotene intake	Inverse variance weighted	7.885002	15	0.92831
Iron intake	Inverse variance weighted	8.512539	12	0.743905
Calcium intake	Inverse variance weighted	17.08543	18	0.517239
Folate intake	Inverse variance weighted	10.30364	13	0.668936
Vitamin B6 intake	Inverse variance weighted	29.46235	15	0.014015
Vitamin B12 intake	Inverse variance weighted	11.05317	8	0.198705
Vitamin E intake	Inverse variance weighted	8.935094	10	0.538274
Omega-3 status	Inverse variance weighted	55.5208	48	0.212415
Omega-6 status	Inverse variance weighted	49.6739	52	0.565885
Omega-7, omega-9, and saturated fatty acids	Inverse variance weighted	4.946173	6	0.550735
Eicosapentaenoic acid status	Inverse variance weighted	3.804596	6	0.7031
Docosahexaenoic acid status	Inverse variance weighted	38.35537	34	0.278503
Years of schooling	Inverse variance weighted	350.4869	304	0.034102
Stress owing to financial difficulties	Inverse variance weighted	93.12477	85	0.256205
Anxiety disorder	Inverse variance weighted	17.94802	16	0.326949
Depression	Inverse variance weighted	39.09844	30	0.123539
Sleep duration	Inverse variance weighted	80.06582	68	0.15022
Night shift work	Inverse variance weighted	2.336928	6	0.886253
Snoring	Inverse variance weighted	40.97262	40	0.427655
Insomnia	Inverse variance weighted	40.6328	38	0.355157
Years of smoking	Inverse variance weighted	6.706376	9	0.667659
Heavy physical activity	Inverse variance weighted	22.71448	16	0.121583
Light physical activity	Inverse variance weighted	11.17844	11	0.428438
Number of days/weeks of vigorous	Inverse variance weighted	8.091923	10	0.619858

physical activity				
Number of days/weeks of moderate physical activity	Inverse variance weighted	12.16205	17	0.790226
Body mass index	Inverse variance weighted	446.0425	431	0.29835
Waist-to-hip ratio	Inverse variance weighted	21.31314	29	0.847488
Fasting glucose	Inverse variance weighted	32.55715	29	0.295958
Fasting proinsulin	Inverse variance weighted	6.977267	7	0.431251
Hemoglobin A1c	Inverse variance weighted	19.5097	10	0.034246
Systolic blood pressure	Inverse variance weighted	469.7801	436	0.12754
Diastolic blood pressure	Inverse variance weighted	497.6532	436	0.021736
High density lipoprotein	Inverse variance weighted	351.3996	325	0.15041
Low density lipoprotein	Inverse variance weighted	161.4808	157	0.386657
Total cholesterol	Inverse variance weighted	95.68587	83	0.161175
Total triglycerides	Inverse variance weighted	14.90952	11	0.186677
Alcohol intake frequency	MR Egger	53.35648	40	0.076905
Alcoholic drinks per week	MR Egger	26.36824	32	0.747092
Average weekly red wine intake	MR Egger	18.34481	16	0.304096
Coffee intake	MR Egger	38.26757	36	0.366894
Decaffeinated coffee intake	MR Egger	22.75442	12	0.029885
Carbohydrate	MR Egger	9.583448	17	0.920164
Process meat	MR Egger	29.51307	21	0.102218
Cheese intake	MR Egger	67.84039	59	0.201272
Saturated fat intake	MR Egger	4.620881	9	0.866027
Polyunsaturated fat intake	MR Egger	16.22174	13	0.237363
Total sugar intake	MR Egger	12.15255	16	0.733413
Blood methionine	MR Egger	8.608551	11	0.657974
Blood selenium	MR Egger	7.938914	10	0.634804
Blood zinc	MR Egger	8.121114	5	0.149686
Vitamin D intake	MR Egger	13.62683	12	0.32517
Carotene intake	MR Egger	7.810158	14	0.89898
Iron intake	MR Egger	8.38207	11	0.678725
Calcium intake	MR Egger	14.40562	17	0.638211
Folate intake	MR Egger	10.22226	12	0.596469
Vitamin B6 intake	MR Egger	27.59448	14	0.016097
Vitamin B12 intake	MR Egger	8.59822	7	0.282803
Vitamin E intake	MR Egger	7.664051	9	0.568324
Omega-3 status	MR Egger	54.48401	47	0.211179
Omega-6 status	MR Egger	49.42193	51	0.536513
Omega-7, omega-9, and saturated fatty acids	MR Egger	3.278916	5	0.657071
Eicosapentaenoic acid status	MR Egger	3.105548	5	0.683717
Docosahexaenoic acid status	MR Egger	38.35379	33	0.239619
Years of schooling	MR Egger	350.3478	303	0.031571

Stress owing to financial difficulties	MR Egger	92.5276	84	0.245749
Anxiety disorder	MR Egger	16.9909	15	0.319409
Depression	MR Egger	37.93722	29	0.123739
Sleep duration	MR Egger	78.16502	67	0.165406
Night shift work	MR Egger	2.306886	5	0.805255
Snoring	MR Egger	40.50421	39	0.403803
Insomnia	MR Egger	38.64267	37	0.395306
Years of smoking	MR Egger	6.29825	8	0.613862
Heavy physical activity	MR Egger	22.71417	15	0.0904
Light physical activity	MR Egger	11.15277	10	0.34574
Number of days/weeks of vigorous physical activity	MR Egger	8.069782	9	0.527129
Number of days/weeks of moderate physical activity	MR Egger	11.59868	16	0.771114
Body mass index	MR Egger	441.4253	430	0.341312
Waist-to-hip ratio	MR Egger	21.23591	28	0.815377
Fasting glucose	MR Egger	30.34784	28	0.346786
Fasting proinsulin	MR Egger	6.598684	6	0.359559
Hemoglobin A1c	MR Egger	19.09216	9	0.024421
Systolic blood pressure	MR Egger	469.5703	435	0.122047
Diastolic blood pressure	MR Egger	496.7136	435	0.021529
High density lipoprotein	MR Egger	351.073	324	0.14428
Low density lipoprotein	MR Egger	160.9219	156	0.376978
Total cholesterol	MR Egger	91.75238	82	0.216265
Total triglycerides	MR Egger	14.42383	10	0.154522

eTable 6. Weighted median analysis of risk factors of MwA

Exposure	No. of SNP	Beta	se	P-value	OR	95%CI lower bound	95%CI upper bound
Alcohol intake frequency	42	0.091481	0.235491	0.69767	1.095796	0.690678	1.738534
Alcoholic drinks per week	34	-0.2259	0.443892	0.610825	0.797802	0.334231	1.904336
Average weekly red wine intake	18	-0.80541	0.692199	0.244607	0.446906	0.115081	1.735513
Coffee intake	38	-0.71041	0.453556	0.117275	0.491442	0.202022	1.195492
Decaffeinated coffee intake	14	-0.1154	0.548614	0.833392	0.891007	0.304013	2.611384
Carbohydrate	19	-0.18732	0.329867	0.570122	0.829176	0.434369	1.582834
Process meat	23	-0.6163	0.555629	0.267347	0.539938	0.181712	1.604374
Cheese intake	61	-0.1531	0.349091	0.660966	0.85804	0.432868	1.700827
Saturated fat intake	11	0.159508	0.417188	0.702208	1.172934	0.517792	2.656999
Polyunsaturated fat intake	15	-0.12369	0.290373	0.670117	0.883649	0.500161	1.56117
Total sugar intake	18	-0.05913	0.302817	0.845192	0.942587	0.520666	1.706413
Blood methionine	13	0.115562	1.426153	0.935417	1.122505	0.068584	18.37189
Blood selenium	12	-0.19813	3.566065	0.955692	0.820262	0.000756	890.1207
Blood zinc	7	0.013289	0.078615	0.865765	1.013378	0.868667	1.182196
Vitamin D intake	14	9.892217	5.346817	0.064297	19775.85	0.55569	7.04E+08
Carotene intake	16	-0.16341	0.3163	0.605406	0.84924	0.456867	1.578594
Iron intake	13	0.039984	0.382766	0.916804	1.040794	0.491528	2.203847
Calcium intake	19	-0.11841	0.3146	0.706643	0.888336	0.479495	1.645773
Folate intake	14	0.422107	0.296076	0.153963	1.525172	0.853678	2.724857
Vitamin B6 intake	16	-0.0348	0.361364	0.923275	0.965796	0.475648	1.961033
Vitamin B12 intake	9	0.03462	0.381568	0.927707	1.035226	0.490048	2.186916
Vitamin E intake	9	0.03462	0.381568	0.927707	1.035226	0.490048	2.186916
Omega-3 status	49	0.063867	0.069497	0.3581	1.065951	0.930208	1.221501
Omega-6 status	53	0.019915	0.124463	0.872873	1.020115	0.79929	1.301948
Omega-7, omega-9, and saturated fatty acids	7	0.090905	0.127581	0.476135	1.095165	0.852866	1.406302
Eicosapentaenoic acid status	7	0.854388	0.586522	0.145199	2.349936	0.744386	7.418457
Docosahexaenoic acid status	35	-0.15436	0.362906	0.67058	0.856961	0.420774	1.745311
Years of schooling	305	-0.30769	0.184873	0.096047	0.735143	0.511688	1.056183
Stress owing to financial difficulties	86	1.183122	1.242617	0.341036	3.264549	0.285816	37.28723
Anxiety disorder	17	-17.8656	12.30314	0.146469	1.74E-08	5.87E-19	517.2543
Depression	31	4.585622	2.627691	0.080965	98.06412	0.568557	16913.99
Sleep duration	69	-0.24387	0.427324	0.56821	0.78359	0.339112	1.810648
Night shift work	7	-0.16001	0.419826	0.703096	0.852131	0.374234	1.940305

Snoring	41	1.702968	0.92506	0.065632	5.490219	0.895701	33.65242
Insomnia	39	0.259164	0.638205	0.684682	1.295846	0.370939	4.526938
Years of smoking	10	0.229054	0.334519	0.493517	1.25741	0.652722	2.422286
Heavy physical activity	17	-0.61112	1.501521	0.684006	0.542741	0.028607	10.29706
Light physical activity	12	-0.9735	1.596461	0.542001	0.377757	0.01653	8.632704
Number of days/weeks of vigorous physical activity	11	0.220891	0.40058	0.581341	1.247187	0.568789	2.734713
Number of days/weeks of moderate physical activity	18	0.036407	0.28679	0.898982	1.037078	0.591141	1.819416
Body mass index	432	-0.16573	0.121881	0.173891	0.847271	0.66723	1.075894
Waist-to-hip ratio	30	-0.43842	0.290859	0.131727	0.645054	0.364764	1.140725
Fasting glucose	30	0.735503	0.305492	0.016058	2.08653	1.146528	3.797212
Fasting proinsulin	8	-0.11859	0.167777	0.479657	0.888168	0.639265	1.233985
Hemoglobin A1c	11	0.926465	0.419042	0.027042	2.525566	1.11087	5.74188
Systolic blood pressure	437	0.009357	0.007547	0.215038	1.009401	0.99458	1.024442
Diastolic blood pressure	437	0.018659	0.012239	0.127354	1.018834	0.994686	1.043569
High density lipoprotein	326	-0.11625	0.104721	0.26696	0.890252	0.725058	1.093085
Low density lipoprotein	158	-0.08025	0.111111	0.470142	0.922886	0.74228	1.147434
Total cholesterol	84	0.003339	0.101503	0.973756	1.003345	0.822335	1.224198
Total triglycerides	12	0.055942	0.101394	0.581134	1.057536	0.866936	1.290042

eTable 7. MR-Egger analysis of risk factors of MwA

Exposure	No. of SNP	Beta	se	P-value	OR	95%CI lower bound	95%CI upper bound
Alcohol intake frequency	42	0.340566	0.451461	0.455049	1.405744	0.580249	3.405633
Alcoholic drinks per week	34	-0.38926	0.768799	0.616103	0.677559	0.150152	3.057472
Average weekly red wine intake	18	-2.63526	2.606404	0.32703	0.071701	0.000433	11.86148
Coffee intake	38	-0.60255	0.586955	0.311471	0.547413	0.173256	1.729584
Decaffeinated coffee intake	14	-0.48368	0.720924	0.514979	0.61651	0.150064	2.532811
Carbohydrate	19	0.434101	0.741906	0.566156	1.543575	0.360582	6.60771
Process meat	23	-0.01281	2.239258	0.995491	0.987276	0.012256	79.53044
Cheese intake	61	-0.47306	1.194192	0.693433	0.62309	0.059984	6.472441
Saturated fat intake	11	0.96017	0.90286	0.315266	2.612141	0.445109	15.32944
Polyunsaturated fat intake	15	-0.13355	0.387357	0.73577	0.874979	0.409518	1.869487
Total sugar intake	18	0.309186	0.448424	0.50039	1.362315	0.565681	3.280828
Blood methionine	13	-0.35897	3.623797	0.922873	0.698395	0.000575	848.6699
Blood selenium	12	22.12363	59.5043	0.7178	4.06E+09	9.06E-42	1.82E+60
Blood zinc	7	0.552969	0.229192	0.060663	1.738407	1.109325	2.724231
Vitamin D intake	14	25.41854	17.01139	0.160945	1.09E+11	0.000362	3.31E+25
Carotene intake	16	-0.33438	0.528937	0.537459	0.71578	0.253828	2.018461
Iron intake	13	-0.5153	0.82958	0.547152	0.597321	0.117505	3.036413
Calcium intake	19	-0.27272	0.963036	0.780453	0.761306	0.115294	5.027035
Folate intake	14	0.194705	0.422511	0.653163	1.214952	0.530775	2.781042
Vitamin B6 intake	16	-0.38269	0.59052	0.527439	0.682027	0.214358	2.170014
Vitamin B12 intake	9	0.600119	0.688389	0.412232	1.822336	0.472781	7.0242
Vitamin E intake	9	0.600119	0.688389	0.412232	1.822336	0.472781	7.0242
Omega-3 status	49	0.06789	0.087177	0.440022	1.070248	0.902148	1.26967
Omega-6 status	53	-0.19595	0.177303	0.274269	0.822052	0.580732	1.163651
Omega-7, omega-9, and saturated fatty acids	7	0.566622	0.400208	0.215994	1.762304	0.804298	3.861398
Eicosapentaenoic acid status	7	1.591766	1.307155	0.277652	4.912417	0.378986	63.67483
Docosahexaenoic acid status	35	0.750011	0.700426	0.292032	2.117023	0.536428	8.354875
Years of schooling	305	0.174072	0.469135	0.710861	1.190141	0.474528	2.984932
Stress owing to financial difficulties	86	-3.43619	2.573647	0.185437	0.032187	0.000207	4.993625

Anxiety disorder	17	-37.6927	19.31264	0.069901	4.27E-17	1.55E-33	1.173559
Depression	31	6.512701	5.238197	0.223712	673.6437	0.02342	19376417
Sleep duration	69	2.350212	1.156892	0.046175	10.48779	1.08622	101.2629
Night shift work	7	0.442243	1.166277	0.720115	1.556193	0.158237	15.30448
Snoring	41	0.867606	3.67987	0.814844	2.381203	0.001756	3229.722
Insomnia	39	0.362131	1.234403	0.770882	1.436387	0.127799	16.14419
Years of smoking	10	0.198676	0.733416	0.793333	1.219786	0.289726	5.135468
Heavy physical activity	17	7.530276	7.901219	0.35567	1863.62	0.000351	9.91E+09
Light physical activity	12	3.209882	4.744498	0.514036	24.77615	0.002267	270789.6
Number of days/weeks of vigorous physical activity	11	1.0782	2.65299	0.693936	2.939384	0.016218	532.7539
Number of days/weeks of moderate physical activity	18	1.206544	1.321189	0.37468	3.341913	0.250828	44.52606
Body mass index	432	-0.39616	0.218606	0.070652	0.6729	0.438399	1.032837
Waist-to-hip ratio	30	-0.65746	0.96259	0.50021	0.518164	0.078541	3.418537
Fasting glucose	30	-0.35147	0.482828	0.472698	0.703655	0.273129	1.812806
Fasting proinsulin	8	0.235511	0.31001	0.476221	1.265555	0.689279	2.323631
Hemoglobin A1c	11	-1.26642	0.802096	0.148817	0.281838	0.058511	1.357554
Systolic blood pressure	437	0.01125	0.012791	0.379583	1.011314	0.986275	1.036988
Diastolic blood pressure	437	0.011249	0.020191	0.577747	1.011312	0.972071	1.052138
High density lipoprotein	326	-0.06958	0.09024	0.441215	0.932782	0.781567	1.113254
Low density lipoprotein	158	-0.01236	0.112979	0.91305	0.98772	0.791524	1.232547
Total cholesterol	84	-0.04891	0.109995	0.65771	0.952263	0.767586	1.181371
Total triglycerides	12	0.036953	0.214638	0.866744	1.037644	0.68131	1.580345

eTable 8. IVW Radial analysis (MwA)

Exposure	No. of SNP	Beta	se	P-value	OR	95%CI lower bound	95%CI upper bound
Alcohol intake frequency	42	-0.04067	0.132961	0.759689	0.960145	0.739874	1.245992
Alcoholic drinks per week	34	-0.06025	0.327978	0.854241	0.941526	0.495053	1.790659
Average weekly red wine intake	18	-0.68467	0.533209	0.199119	0.504254	0.177326	1.433924
Coffee intake	38	-0.93305	0.288312	0.001211	0.393352	0.223545	0.692146
Decaffeinated coffee intake	14	-0.07827	0.401695	0.84552	0.924719	0.420804	2.032074
Carbohydrate	19	-0.00817	0.270878	0.975924	0.991858	0.583276	1.686652
Process meat	23	-0.23757	0.435902	0.585744	0.78854	0.335565	1.85298
Cheese intake	61	-0.07756	0.280614	0.782247	0.925372	0.533892	1.603908
Saturated fat intake	11	0.006641	0.307005	0.982743	1.006663	0.551514	1.837434
Polyunsaturated fat intake	15	-0.36082	0.20034	0.071699	0.697107	0.470725	1.032362
Total sugar intake	18	-0.09917	0.211876	0.63973	0.905585	0.597829	1.371772
Blood methionine	13	-0.30644	0.683683	0.653994	0.736062	0.192731	2.811107
Blood selenium	12	-2.10971	2.825766	0.455306	0.121273	0.000477	30.83919
Blood zinc	7	-0.02398	0.076034	0.752473	0.976306	0.841133	1.133201
Vitamin D intake	14	4.180866	3.85143	0.277684	65.42247	0.034461	124202.5
Carotene intake	16	-0.10434	0.239955	0.663695	0.900922	0.562903	1.441919
Iron intake	13	-0.33376	0.302907	0.270525	0.716226	0.395559	1.29685
Calcium intake	19	0.047041	0.245274	0.847907	1.048165	0.648109	1.695162
Folate intake	14	0.202591	0.215931	0.348131	1.224571	0.80201	1.86977
Vitamin B6 intake	16	-0.1764	0.25008	0.480576	0.838283	0.513474	1.368556
Vitamin B12 intake	9	0.320575	0.231716	0.166517	1.37792	0.87495	2.170026
Vitamin E intake	11	-0.25844	0.353051	0.464154	0.772254	0.386578	1.542707
Omega-3 status	49	0.021944	0.062357	0.724913	1.022186	0.904587	1.155073
Omega-6 status	53	-0.07729	0.087631	0.377766	0.925619	0.779541	1.099071
Omega-7, omega-9, and saturated fatty acids	7	0.054453	0.089116	0.541175	1.055963	0.886731	1.257494
Eicosapentaenoic acid status	7	0.932142	0.283187	0.000996	2.539943	1.458042	4.424638
Docosahexaenoic acid status	35	0.074196	0.285711	0.795103	1.077018	0.615206	1.885494
Years of schooling	305	-0.14917	0.115723	0.197385	0.861422	0.686611	1.08074
Stress owing to financial difficulties	86	1.226929	0.868299	0.157648	3.410739	0.621924	18.70508
Anxiety disorder	17	-6.25401	9.672942	0.517925	0.001923	1.12E-11	329375.6
Depression	31	2.669394	1.520228	0.079103	14.43122	0.733261	284.0191
Sleep duration	69	-0.10856	0.271682	0.689462	0.897125	0.526736	1.527964
Night shift work	7	0.22127	0.35181	0.529385	1.24766	0.626079	2.486356
Snoring	41	0.926303	0.690314	0.179642	2.525158	0.652652	9.770022
Insomnia	39	0.198589	0.419304	0.635775	1.21968	0.5362	2.774372
Years of smoking	10	0.400482	0.297959	0.178921	1.492544	0.832337	2.676423

Heavy physical activity	17	-0.61365	1.260779	0.626456	0.541373	0.04574	6.407568
Light physical activity	12	-2.43195	1.210961	0.044614	0.087866	0.008185	0.943214
Number of days/weeks of vigorous physical activity	11	0.077728	0.320091	0.808135	1.080829	0.577152	2.024061
Number of days/weeks of moderate physical activity	18	-0.31888	0.214561	0.137227	0.726963	0.477391	1.107007
Body mass index	432	-0.10026	0.080609	0.213593	0.904605	0.772402	1.059435
Waist-to-hip ratio	30	-0.21365	0.216098	0.322818	0.807629	0.528769	1.233554
Fasting glucose	30	0.101304	0.226524	0.654722	1.106613	0.709863	1.725111
Fasting proinsulin	8	-0.03499	0.114473	0.759858	0.965615	0.771547	1.208495
Hemoglobin A1c	11	0.318923	0.367011	0.384862	1.375646	0.670039	2.824315
Systolic blood pressure	437	0.008096	0.005082	0.111133	1.008129	0.998137	1.018221
Diastolic blood pressure	437	0.011514	0.008313	0.166005	1.011581	0.995233	1.028198
High density lipoprotein	326	-0.10304	0.059217	0.081844	0.902089	0.803236	1.013108
Low density lipoprotein	158	-0.09594	0.077113	0.21345	0.90852	0.781079	1.056754
Total cholesterol	84	-0.0122	0.067199	0.855989	0.987879	0.86597	1.12695
Total triglycerides	12	0.049566	0.071933	0.490789	1.050815	0.912632	1.20992

eTable 9. Heterogeneity test (MwA)

Exposure	method	Q-value	Q_df	P-value
Alcohol intake frequency	Inverse variance weighted	29.2125	41	0.915762
Alcoholic drinks per week	Inverse variance weighted	37.96156	33	0.253425
Average weekly red wine intake	Inverse variance weighted	22.28887	17	0.173863
Coffee intake	Inverse variance weighted	36.16027	37	0.508226
Decaffeinated coffee intake	Inverse variance weighted	14.94853	13	0.310577
Carbohydrate	Inverse variance weighted	27.41909	18	0.071463
Process meat	Inverse variance weighted	25.72741	22	0.26357
Cheese intake	Inverse variance weighted	91.23073	60	0.005769
Saturated fat intake	Inverse variance weighted	10.66286	10	0.38438
Polyunsaturated fat intake	Inverse variance weighted	12.08539	14	0.599445
Total sugar intake	Inverse variance weighted	18.77713	17	0.341473
Blood methionine	Inverse variance weighted	4.591952	12	0.97024
Blood selenium	Inverse variance weighted	15.3491	11	0.167066
Blood zinc	Inverse variance weighted	9.607043	6	0.142206
Vitamin D intake	Inverse variance weighted	14.9847	13	0.308309
Carotene intake	Inverse variance weighted	15.83885	15	0.392842
Iron intake	Inverse variance weighted	14.31442	12	0.281082
Calcium intake	Inverse variance weighted	22.18641	18	0.223814
Folate intake	Inverse variance weighted	12.39652	13	0.495428
Vitamin B6 intake	Inverse variance weighted	16.98509	15	0.319757
Vitamin B12 intake	Inverse variance weighted	5.022659	8	0.755152
Vitamin E intake	Inverse variance weighted	23.01984	10	0.010674
Omega-3 status	Inverse variance weighted	63.6425	48	0.064699
Omega-6 status	Inverse variance weighted	64.267	52	0.11826
Omega-7, omega-9, and saturated fatty acids	Inverse variance weighted	4.866456	6	0.561052
Eicosapentaenoic acid status	Inverse variance weighted	2.270688	6	0.893197
Docosahexaenoic acid status	Inverse variance weighted	48.15064	34	0.054636
Years of schooling	Inverse variance weighted	282.9135	304	0.801943
Stress owing to financial difficulties	Inverse variance weighted	104.6123	85	0.073225
Anxiety disorder	Inverse variance weighted	19.3802	16	0.249441
Depression	Inverse variance weighted	19.66773	30	0.924907
Sleep duration	Inverse variance weighted	58.65464	68	0.783315
Night shift work	Inverse variance weighted	9.749629	6	0.135599
Snoring	Inverse variance weighted	52.38347	40	0.090806
Insomnia	Inverse variance weighted	44.58331	38	0.214469
Years of smoking	Inverse variance weighted	11.86214	9	0.221193
Heavy physical activity	Inverse variance weighted	22.28939	16	0.134091
Light physical activity	Inverse variance weighted	12.13179	11	0.353819
Number of days/weeks of vigorous	Inverse variance weighted	12.23356	10	0.269729

physical activity				
Number of days/weeks of moderate physical activity	Inverse variance weighted	18.63052	17	0.350148
Body mass index	Inverse variance weighted	479.5209	431	0.052951
Waist-to-hip ratio	Inverse variance weighted	33.41923	29	0.261154
Fasting glucose	Inverse variance weighted	36.99278	29	0.146409
Fasting proinsulin	Inverse variance weighted	5.401465	7	0.611094
Hemoglobin A1c	Inverse variance weighted	17.31995	10	0.067577
Systolic blood pressure	Inverse variance weighted	548.7154	436	0.000191
Diastolic blood pressure	Inverse variance weighted	515.9399	436	0.004945
High density lipoprotein	Inverse variance weighted	347.9631	325	0.182386
Low density lipoprotein	Inverse variance weighted	196.3763	157	0.018021
Total cholesterol	Inverse variance weighted	106.7175	83	0.040878
Total triglycerides	Inverse variance weighted	9.881199	11	0.541104
Alcohol intake frequency	MR Egger	28.40055	40	0.915019
Alcoholic drinks per week	MR Egger	37.6964	32	0.224825
Average weekly red wine intake	MR Egger	21.50254	16	0.159992
Coffee intake	MR Egger	35.73913	36	0.480899
Decaffeinated coffee intake	MR Egger	14.38791	12	0.276626
Carbohydrate	MR Egger	26.77003	17	0.061528
Process meat	MR Egger	25.71456	21	0.217543
Cheese intake	MR Egger	91.0514	59	0.004673
Saturated fat intake	MR Egger	9.356032	9	0.405079
Polyunsaturated fat intake	MR Egger	11.58442	13	0.56198
Total sugar intake	MR Egger	17.60438	16	0.347567
Blood methionine	MR Egger	4.59172	11	0.949318
Blood selenium	MR Egger	15.09808	10	0.128527
Blood zinc	MR Egger	2.802571	5	0.730391
Vitamin D intake	MR Egger	13.18406	12	0.355806
Carotene intake	MR Egger	15.57013	14	0.340324
Iron intake	MR Egger	14.24207	11	0.219887
Calcium intake	MR Egger	22.03306	17	0.183451
Folate intake	MR Egger	12.39595	12	0.414427
Vitamin B6 intake	MR Egger	16.80478	14	0.26673
Vitamin B12 intake	MR Egger	4.820548	7	0.681852
Vitamin E intake	MR Egger	22.86047	9	0.006517
Omega-3 status	MR Egger	62.87552	47	0.06061
Omega-6 status	MR Egger	63.52689	51	0.11198
Omega-7, omega-9, and saturated fatty acids	MR Egger	3.122049	5	0.681176
Eicosapentaenoic acid status	MR Egger	1.98066	5	0.851815
Docosahexaenoic acid status	MR Egger	46.57595	33	0.058841
Years of schooling	MR Egger	282.4094	303	0.796428

Stress owing to financial difficulties	MR Egger	100.2097	84	0.109566
Anxiety disorder	MR Egger	15.81132	15	0.394701
Depression	MR Egger	19.05042	29	0.919988
Sleep duration	MR Egger	54.02146	67	0.873776
Night shift work	MR Egger	9.671875	5	0.085085
Snoring	MR Egger	52.38312	39	0.074405
Insomnia	MR Egger	44.56036	37	0.183627
Years of smoking	MR Egger	11.72638	8	0.163836
Heavy physical activity	MR Egger	20.77951	15	0.144023
Light physical activity	MR Egger	10.54294	10	0.394217
Number of days/weeks of vigorous physical activity	MR Egger	12.04023	9	0.211051
Number of days/weeks of moderate physical activity	MR Egger	17.16273	16	0.375143
Body mass index	MR Egger	477.1664	430	0.05756
Waist-to-hip ratio	MR Egger	33.15376	28	0.230129
Fasting glucose	MR Egger	35.56238	28	0.154083
Fasting proinsulin	MR Egger	4.47672	6	0.612447
Hemoglobin A1c	MR Egger	11.44714	9	0.2463
Systolic blood pressure	MR Egger	548.6214	435	0.00017
Diastolic blood pressure	MR Egger	515.9397	435	0.004493
High density lipoprotein	MR Egger	347.6988	324	0.174833
Low density lipoprotein	MR Egger	195.0949	156	0.018376
Total cholesterol	MR Egger	106.4852	82	0.035922
Total triglycerides	MR Egger	9.877253	10	0.451328
